# Long-Term Outcome Analysis of Surgically Treated Talus Fractures in a Tertiary Care Hospital

**DOI:** 10.7759/cureus.57918

**Published:** 2024-04-09

**Authors:** Chandan Noel Vincent, Aakash Venkatesan, Dinakar Rai, Arvind Kumar SM

**Affiliations:** 1 Trauma and Orthopaedics, Manchester Royal Infirmary, Manchester, GBR; 2 Trauma and Orthopaedics, University Hospital Llandough, Wales, GBR; 3 Trauma and Orthopaedics, PSG Institute of Medical Sciences and Research, Coimbatore, IND

**Keywords:** avascular necrosis (avn), post-traumatic ankle arthritis, talus body fractures, talus neck fracture, talus

## Abstract

Aim

The present study aims to look at the long-term clinical and radiological outcomes of surgically treated talus fractures. We have compared the outcomes and complications between simple and complex talar fracture patterns. Additionally, patients' ability to return to activity following surgical treatment of these fractures was also analysed.

Materials and methods

Retrospective analysis of surgically treated talus fractures at the PSG Institute of Medical Sciences and Research from 2012 to 2015. The fractures were classified as neck and body fractures. The fractures were classified anatomically (neck and body) based on their severity (simple and complex) fracture patterns. A radiological assessment was done at follow-up to assess for complications of malunion, avascular necrosis (AVN), and arthritis. The outcomes were assessed using the American Orthopaedic Foot and Ankle Society (AOFAS) score and the Maryland foot score (MFS).

Results

Twenty patients were included in the analysis. There were 12 talar neck and eight body fractures, subclassified into simple (10) and complex fracture patterns (10). The surgical approach involved either a medial malleolus osteotomy/via fractured medial malleolus (55%) or a non-osteotomy-based approach (anteromedial (AM)/anterolateral (AL)/combined AM and AL) (45%). The average AOFAS score was 71.34, while the MFS was 74.35. The outcomes were consistently unfavourable for patients with complex fractures with a higher propensity for complications, but no difference was observed when comparing neck and body fractures. There was a 10% incidence of malreduction in the non-osteotomy-based approach group. AVN was found in 35% of cases, and post-traumatic arthritis occurred in 75% of cases during the five-year follow-up period.

Conclusion

The findings of the present study consistently reiterate the propensity for complex talus fractures to develop complications like AVN and post-traumatic arthritis in the long term. This study serves to help predict talus fractures based on their severity, with poor outcomes noted with more complex fracture types. We also advocate a more extensile medial malleolus osteotomy-based approach to better visualise complex body fractures of the talus and obtain more anatomical reduction.

## Introduction

The talus is a crucial bone in the ankle joint that plays a vital role in the three major articulations of the hindfoot: the ankle joint, subtalar joint, and talonavicular joint. Articular cartilage covers the majority of its surface area, which is between 70% and 80% [1]. Despite their importance, talus fractures are relatively rare, comprising only 0.3% of all fractures and approximately 3.4% of reported foot fractures [2]. The most common causes of talus fractures are high-energy motor vehicle accidents and falls from heights. These fractures are broadly classified into two types: neck fractures and body fractures. Innokuchi et al. suggested evaluating the course of the inferior fracture line to differentiate between the two [3]. Neck fractures are further classified according to Hawkins classification, with an increasing risk of avascular necrosis (AVN) as the fracture severity worsens [4].

The blood supply to the talus is unique and predisposes it to a higher risk of AVN following fractures. Since the talus lacks muscle attachments, its blood supply primarily relies on the tarsal canal artery and branches of the posterior tibial artery, including the deltoid branches. Preserving the deltoid branches is crucial, as they can sometimes be the only available blood supply to the body of the talus. The head of the talus receives its blood supply from the sinus tarsi and branches of the dorsal pedis artery [5]. Talus fractures are known to have poor outcomes, especially as the severity of the injury increases. The reported rates of AVN range from 27% to 50% in various studies. The only identified risk factor for AVN is the degree of initial displacement observed on CT scans and radiographs. Post-traumatic arthritis is a common long-term complication, with incidence rates ranging from 34% to 100% in studies with longer follow-ups [6].

The present study aims to examine the long-term clinical and radiological outcomes of surgically treated talus fractures. The study also aims to assess outcomes and complications related to the timing of surgery and surgical approaches. Furthermore, the study investigates how outcomes vary with the severity of fracture patterns. Additionally, the researchers analysed whether patients were able to return to their previous level of activity following surgical treatment of these fractures.

## Materials and methods

Materials and methods

From 2012 to 2015, talus fractures treated at the PSG Institute of Medical Sciences and Research were analysed retrospectively. We included patients who underwent open reduction and internal fixation of talus fractures. The research was approved by the institution's ethical committee.

All patients between the ages of 18 and 75 with displaced neck and body talus fractures requiring surgical stabilisation were included in the study. Patients with open fractures, conservatively managed talus fractures, prior foot surgery, rheumatic/inflammatory joint disorders, alcoholism, and peripheral vascular disease were excluded from the study.

Data collection

The authors retrospectively collected data using the patient documents in the hospital's information system. Each patient's trauma profile was obtained, including general demographics, the site of injury, the mechanism of injury, and concomitant injuries. The pre-operative radiographs and CT imaging analysis were used to classify patients into two groups: neck and body fractures. Talar neck fractures were further classified according to the Hawkins classification, whereas talar body fractures were classified according to the Sneppen classification. The patients were further subclassified into simple and complex fractures based on the fracture patterns. The Hawkins 1 and 2 and Sneppen 2, 3, and 5 fractures were grouped into simple fracture patterns. The Hawkins 3 and 4 and Sneppen 6 fracture patterns were grouped into complex fracture patterns. Each patient's treatment profile, including time to surgery, surgical approach, and implants used, was gathered.

During the initial period of follow-up, wound dehiscence, implant irritations, stiffness, infection, and skin necrosis were recorded as acute complications. Patients were evaluated with serial follow-up radiographs to assess for the presence of Hawkin's signs, AVN, and time to union of fractures. The last follow-up radiographs were evaluated for signs of late complications, such as collapse and post-traumatic arthritis. The patient's functional outcomes were evaluated using the American Orthopaedic Foot and Ankle Society (AOFAS) score and the Maryland foot score (MFS).

## Results

Demographics

The study included 20 patients who received surgical fixation of closed talus fractures. There were seven females and 13 males in all. The patients' ages, which ranged from 23 to 71, were 38 years on average. In 12 patients, a motor vehicle collision accounted for 60% of the injuries, while falls from height occurred in eight patients, accounting for 40%. The interval between damage and definitive fixation was 2-20 days, with an average interval of 7.7 days. The fractures were treated acutely in 65% (13 patients) of cases and delayed in 35% (seven patients).

Results and assessment

**Figure 1:**
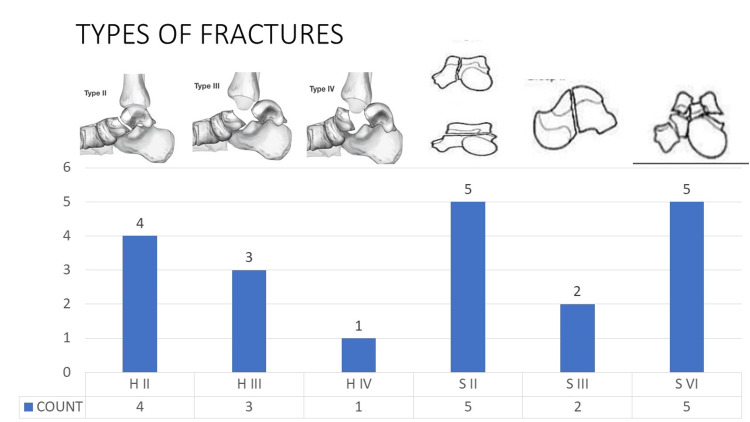
Graph depicting the distribution of fracture variants

To ascertain the type of fracture, pre-operative radiographs and CT scans were evaluated. Eight fractures were classified as neck of talus fractures (40%) and 12 fractures were classified as body of talus fractures (60%) using Inokuchi et al.'s classification system [3]. The Hawkins classification was used to further subclassify the neck of talus fractures based on severity. Hawkins type 2 (H 2) - 4, Hawkins type 3 (H 3) - 3, and Hawkins type 4 (H 4) - 1 were present. The Sneppen classification was used to sub-classify the body of talus fractures based on severity. Sneppen type 2 (S 2): 4, Sneppen type 3 (S 3): 3, and Sneppen type 6 (S 6) - 5 were present (Figure 1).

Based on the fracture patterns, severity, comminution, and displacement, the patients were further divided into simple and complicated fractures. The simple fracture and complex fracture categories each contained ten (50% of the total) patients.

**Figure 2:**
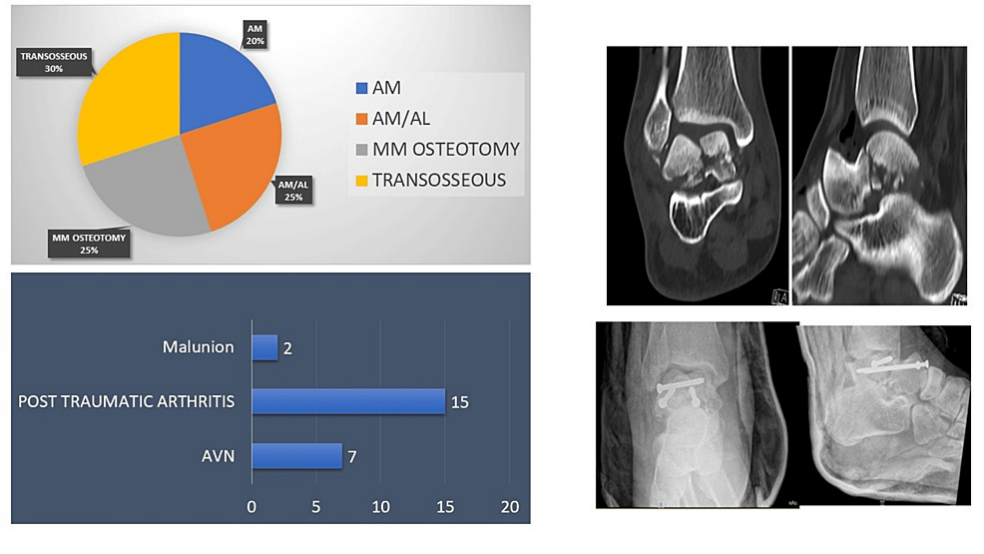
Approaches and complications AM: anteromedial, AL: anterolateral, MM: medial malleolus, AVN: avascular necrosis

In all cases, fractures were repaired with Herbert or cancellous screws; no plates were used. Anteromedial (AM): 20% (four patients); combined (anteromedial and anterolateral (AM and AL)): 25% (five patients); and medial malleolus osteotomy: 25% (five patients) were the approaches utilised. In 30% (six patients), a medial malleolus fracture was present; hence, it was approached through the fractured medial malleolus (Figure 2).

**Figure 3:**
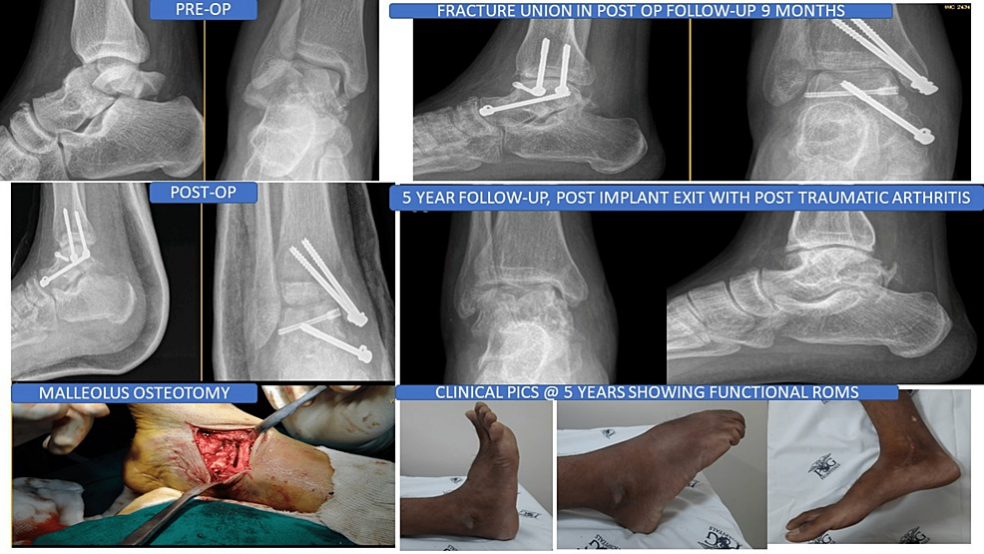
Case illustration with five years of follow-up results

All patients were radiographed at standard intervals (6, 12, and 20 weeks) to assess the presence of Hawkins sign and talus fracture union. In our present case series, no patient failed to achieve radiological union. The average time to radiological union was three months (range: 1.5-5 months). Figure 1 demonstrates that there were two cases of malunion (10%) due to inadequate fracture reduction, with poor outcome scores at follow-up. A Hawkins sign was observed in 35% (seven patients), but these individuals did not develop AVN during subsequent follow-ups. On follow-up radiographs, 13 patients lacked the Hawkins sign; however, AVN developed in only 35% (seven patients). Seventy-five percent (15 patients) developed post-traumatic arthritis after an average of five years of follow-up (Figure 3).

Functional outcomes

The functional outcomes of all 20 patients were evaluated using the AOFAS ankle-hind foot score and the MFS. The average AOFAS score was 71.34 (range: 25 to 93). Twenty percent of cases (four) had an excellent AOFAS score, 35% of cases (seven) had a good outcome, 30% of cases (six) had a fair outcome, and 15% of cases (three) had a poor outcome. The average MFS was 74.35 (range: 22-98). Twenty-five (five cases) MFS were excellent, 40% (eight cases) were good, 25% (five cases) were average, and 10% (two cases) were poor.

The independent student t-test was utilised to assess for any statistically significant differences in outcome scores between the neck and body fracture groups. The average AOFAS score for the neck fracture group was 63.25 +/- 21.98, while the average score for the body fracture group was 70.58 +/- 17. The average MFS score for the neck fracture group was 68.25 +/- 23.67, while the average MFS score for the body fracture group was 73.42 +/- 18. Comparing the AOFAS and MFS for fractures of the neck and body revealed no statistically significant differences (Table 1).

**Table 1:** Neck versus body fractures of the talus comparison AOFAS: American Orthopaedic Foot and Ankle Society, MFS: Maryland foot score, AVN: avascular necrosis

AOFAS and MFS			
	Neck	Body	
AOFAS	63.25	76.58	Statistically not significant
MAFS	68.25	78.42	
Incidence of AVN - neck versus body fractures			
	Neck	Body	
Presence of AVN	37.50% (3)	33.30% (4)	Statistically not significant
Incidence of post-traumatic arthritis - neck versus body fractures			
	PTA		
	Neck	Body	
Neck fractures	87.50% (7)	66.70% (8)	Statistically not significant

A student t-test was used to determine whether there were any statistically significant differences in outcome scores between the simple fracture and complex fracture groups. The average AOFAS score for simple fractures was 80.40 +/- 15.24, and for complex fractures, it was 62.10 +/- 20.69. Comparing the AOFAS scores of the groups with simple and complex fractures yielded a statistically significant difference (p. 149). The average MFS score for the simple fracture group was 85.40 +/- 14.43, and for the complex fracture group, it was 63.30 +/- 21.22. Comparing the Maryland foot score between simple and complex fractures revealed a statistically significant difference (p. 301) (Table 2).

**Table 2:** Simple (10, 50%) versus complex (10, 50%) fractures AOFAS: American Orthopaedic Foot and Ankle Society, MFS: Maryland foot score

AOFAS and MFS - simple versus complex fractures				
Outcome scores	Simple	Complex		
AOFAS	80.4	62.1	Statistically significant	
MAFS	85.4	63.3		
Incidence of post-traumatic arthritis – simple versus complex fractures				
	Simple	Complex	p-value	
Presence of post-traumatic arthritis	60% (6)	90% (9)	0.12	Statistically significant

Complications

In the present investigation, the incidence of AVN was 35% overall. There was no statistically significant difference between early and delayed fixation in the incidence of AVN (p=0.720) (Table 3). The incidence of AVN was 37.50% in the group with neck fractures and 33.30% in the group with body fractures. Comparing the incidence of AVN between groups with neck and body fractures revealed no difference. Between the two groups, there was no statistically significant difference in the incidence of AVN (p=0.848). During the long-term follow-up, the incidence of post-traumatic arthritis was 87.50% in the group with neck fractures and 66.70% in the group with body fractures. This difference was statistically significant, however.

**Table 3:** Early versus delayed surgery AVN: avascular necrosis

		No complications	Other complications	AVN	p-value
Early	Count	3	5	5	0.72
	Percentage	23.10%	38.50%	38.50%	
Delayed	Count	1	4	2	
	Percentage	14.30%	57.10%	28.60%	
Total	Count	4	9	7	
	Percentage	20%	45%	35%	

Mal-union of the fracture was reported in 10% (two cases) due to improper reduction, resulting in poor outcomes in both cases. In addition, there was a 10% incidence (two cases) of wound dehiscence and a 10% incidence (two cases) of implant-related irritation that required removal. Early versus delayed definitive fixation did not affect outcome scores or complication rates. At the final follow-up, a straightforward survey was conducted to determine whether patients were able to return to their pre-injury level of activity and recreational sports. None of the patients in our study were able to return to their pre-injury level or recreational sports. Pain, apprehension, and rigidity were the most frequently cited factors (Figure 1).

## Discussion

In the present study of 20 patients, the incidence of AVN was 35% (seven patients), with no patients experiencing catastrophic collapse. A meticulous reduction of the fracture may probably aid in the revascularization of these fractures in the long term. This may explain why none of these fractures experienced a catastrophic failure during extended follow-up. The fractures were treated in an average of 7.7 days (7-20 days). After a positive wrinkle sign and improvement of local skin conditions, the surgery was performed immediately in 65% of patients and delayed in 35% of patients. Notable is the fact that some of these patients presented late following initial treatment with conventional bone setters. We found no statistically significant difference between early and delayed fracture fixation in the incidence of AVN.

Fifty-five percent of the patients in the present study required an osteotomy-based approach, or the fractures were approached through an already fractured medial malleolus. Forty-five percent had a single or combined (AM and AL) approach to fix the fracture. Overall, there were only two cases of wound dehiscence and superficial infection; we did not encounter any deep infection or skin necrosis in the present study. We did have two cases of mal-reduction of body fractures (10%), and both of these occurred in the non-osteotomy groups. Both of these cases had poor outcomes at long-term follow-up. Magnusson et al., in a cadaveric study, compared an extensile posteromedial approach to a medial malleolus osteotomy approach to visualise the body of the talus. A surface area of 11.2 cm2 was visualised with a medial malleolus osteotomy, whereas only 6.7 cm2 was visualised with a posteromedial approach without osteotomy. They were able to enhance visualisation with gastrocnemius recession and distraction of the joint [7]. The bottom line is to adequately visualise the body fractures, and attaining anatomical reduction is paramount. This can be adequately done with the medial malleolus osteotomy approach. Giordano et al., in a meta-analysis, noted that the that the adequacy of fracture reduction is underreported in talus fractures. They identify only three studies that specifically report the adequacy of fracture reduction, with 10 mal-reduced fractures identified. The risk factors included open talar neck fracture dislocations, articular/neck incongruence with a gap or articular step off >3 mm, angulation more than five degrees, and comminution of the neck [8].

In our present study, the average AOFAS score was 71.34, and the MFS score was 74.35. Talar body fractures (AOFAS = 70.58/MFS = 73.42) had slightly higher outcome scores compared to neck fractures (AOFAS = 63.25/MFS = 68.25). This difference, however, was not statistically significant. Most studies do not compare the outcomes of neck and body fractures directly. We came across a few studies comparing the two groups and found them to have similar results. Ohl et al. found no significant differences between the AOFAS scores between the neck (AOFAS = 64.2) and body fractures of the talus (AOFAS = 69.2) [9]. Lindvall et al. compared AOFAS scores between 16 neck fractures and eight body fractures. They found no significant differences in outcomes. Interestingly, neck fractures with AVN had inferior outcomes compared to those without, whereas there was no difference in outcomes when comparing body fractures with and without AVN [10]. Gomes et al. found inferior outcomes with neck fractures (AOFAE = 61) compared to body fractures (AOFAS = 82) in 11 patients [11]. Biz et al., in their review of 27 patients with talar neck and body fractures, found no statistically significant differences between the outcomes of both groups [12].

Complex fractures of the talus consistently resulted in inferior outcomes. The AOFAS and MFS averages were consistently lower in the group with complex fractures. The prevalence of AVN in the present study was 35%. The incidence of AVN did not differ between the groups with neck and body fractures. The timing of fracture fixation did not appear to influence the development of AVN. The incidence of post-traumatic arthritis of varying severity was 75% at an average follow-up of five years after surgery. The incidence of post-traumatic arthritis was higher among patients with neck fractures (87.5%) than among those with body fractures (66.7%). There was also a correlation between fracture complexity and the incidence of post-traumatic arthritis (90% in the complex fracture group). In a systematic review of 1086 talus neck and body fractures, Wijers et al. reported a prevalence of 29% for AVN and 69% for post-traumatic arthritis [6]. Vints et al. conducted a clinical follow-up of 84 patients who had undergone operative fixation of talus fractures for 9.1 years. This is the only case series with the longest available follow-up, as far as we are aware. They reported reduced rates of arthritis, but the radiologic follow-up period in their study was only three years [13]. We may identify a greater incidence of radiological post-traumatic arthritis with varying degrees of clinical symptomatology if we monitor these fractures radiologically and clinically for an extended period of time.

In the present series, none of the patients were able to return to preinjury functional status. The main drawback is that it is a retrospective review.

## Conclusions

The findings of the present study consistently reiterate the propensity for complex talus fractures to develop complications like AVN and post-traumatic arthritis in the long term. This study serves to help predict talus fractures based on their severity, with poor outcomes noted with more complex fracture types. We also advocate a more extensile medial malleolus osteotomy-based approach to better visualise complex body fractures of the talus and obtain more anatomical reduction.
